# Natural history of Sanfilippo syndrome in Spain

**DOI:** 10.1186/1750-1172-8-189

**Published:** 2013-12-06

**Authors:** Verónica Delgadillo, Maria del Mar O’Callaghan, Laura Gort, Maria Josep Coll, Mercedes Pineda

**Affiliations:** 1 Neuropediatrics Department, Hospital Sant Joan de Déu, Barcelona, Spain; 2 Errores Congénitos del Metabolismo, Hospital Clinic, CIBERER, IDIBAPS, Barcelona, Spain; 3 Fundación Hospital Sant Joan de Déu, CIBERER C-III, Barcelona, Spain

**Keywords:** Mucopolysacharidosis, Natural history, Psychomotor delay, Coarse features, Language delay

## Abstract

**Background:**

Mucopolysaccharidosis type III (MPS III), or Sanfilippo syndrome, is caused by a deficiency in one of the four enzymes involved in the lysosomal degradation of heparan sulphate. Four MPS III types have been recognized, characterized by a large phenotypic heterogeneity. This is the first Spanish study describing the natural history of Sanfilippo patients (MPSIIIA, MPSIIIB and MPSIIIC), representing an essential step for understanding patient prognosis and for the establishment and application of future therapies.

**Methods:**

This retrospective study aimed to establish the natural history of MPS III in Spain based on an extensive chronological data survey involving physicians and parents of 55 Spanish MPSIII patients. In addition to clinical description we report biochemical and molecular analysis already performed in the majority of cases.

**Results:**

The most frequent subtype was MPS IIIA (62%). Symptoms before diagnosis were speech delay in 85%, followed by coarse facial features in 78%, and hyperactivity in 65% of cases at a mean age of 3 years old. The median age at clinical and biochemical diagnosis for each MPS III subtype were as follows: IIIA 4.4 years (1.2 – 16 years), IIIB 3.1 years (1–29 years), and IIIC 6.3 years (3.4-22 years).

45% of patients developed epilepsy at a median age of 8.7 (2.5 – 37) years old.

Age of death for MPS IIIA patients was 15 years (11.5 – 26 years).

Molecular analysis of our cohort reveals, as alluded to above, a great allelic heterogeneity in the three subtypes without clear genotype-phenotype correlations in most cases.

**Conclusion:**

MPS IIIA is the most frequent subtype in Spanish Sanfilippo patients. Diagnosing physicians should consider Sanfilippo syndrome in children with non-specific speech delay, behavioural abnormalities, and/or mild dysmorphic features. We stress the importance of establishing early diagnosis procedures as soon as possible so as to be able to determine future short-term enzymatic or gene therapy treatments that can change the prognosis of the disease.

## Background

Mucopolysaccharidosis type III (MPS III), or Sanfilippo syndrome, is an autosomal recessive disorder caused by a deficiency in one of the four enzymes involved in the lysosomal degradation of heparan sulphate. Based on the relevant enzyme deficiency, four types have been recognized: heparan N-sulphatase is deficient in type A (OMIM #252900), α–N- acetylglucosaminidase in type B (OMIM #252920), acetyl-CoA α-glucosamide acetyltransferase in type C (OMIM# 252930), and N- acetyl glucosamine 6-sulphatase in type D (OMIM# 252940). This disorder primarily affects the central nervous system. All types of MPS III are characterized by progressive mental deterioration and behavioural problems with more or less prominent dysmorphic facial features and mild somatic signs [1].

The clinical evolution of Sanfilippo disease has three stages. After a period of normal development, the first phase usually starts between 1 and 3 years of age with slowed cognitive development, most notably speech delay. The second phase generally starts at around 3–4 years of age with severe behavioural problems and progressive intellectual decline [2-6]. Finally, the third stage usually begins in the teen years with the onset of severe dementia; behavioural problems slowly disappear, and all motor functions start to decline, eventually resulting in complete loss of locomotion, dysphagia, and pyramidal tract lesions [5,6]. Patients usually die at the end of the second or beginning of the third decade of life, although longer survival has been reported in patients with an attenuated phenotype [7-9].

MPS III A has been reported to be the most severe form, with earlier onset and faster progression of symptoms than MPS IIIB and IIIC [3,10,11]. MPS IIID is very rare and heterogeneous [12-14].

Some studies of MPS III are characterized by great phenotypic variability, due to a large allelic heterogeneity with variations in the residual enzymatic activity [15-17]. Over 300 mutations in the four genes encoding for the enzymes have been described to date [5,18]. In most cases no genotype-phenotype correlations can be established. However Meyer [10] and Valstar [7] revealed a significant correlation between the phenotype and genotype in patients affected by MPS IIIA [9,10]. In recent years, several studies from different countries have described the natural course of MPS III [9-12,14,19,20].

This is the first retrospective study to consider extensive clinical histories in a large cohort of Spanish Sanfilippo patients. It is based on a survey involving physicians and parents of MPSIII patients.

We aimed to establish the natural history of MPS III in Spain—something that is essential for the development of future therapies.

## Material and methods

### Patients and data base

In order to obtain a representative sample of Spanish patients affected by Sanfilippo disease, all families of MPS III children registered with the Spanish MPS Association (MPS España) were invited to participate in this study. *We offered to physicians that we knew had patients dead or alive to enter in our study with agreement of their families*. Of 70 information sheets sent out, 55 patient families (78.5%) agreed to enrol in the study. Five families had two children affected by MPS III (three pairs of siblings had IIIA, one pair had IIIB, and one had IIIC).

All patients were diagnosed with MPS III A, B or C on the basis of analysis of urinary GAGs levels, and measurement of enzymatic activities of particular lysosomal hydrolases in leukocytes or skin fibroblasts. In most cases identification of pathogenic mutations was performed. We had no information of any MPS IIID patient alive at the time of the study. The percentages of each subtype of Sanfilippo in this study were MPS IIIA 62%, MPS IIIB 20%, and MPS IIIC 18%.

Data from each patient were collected through a questionnaire answered by their physician and by the parents. This questionnaire was designed to retrospectively assess the information available in the patient medical history and/or parent reports. Furthermore, the families were asked to provide material on the diagnosis and clinical course of the disease (pictures at different ages, school and medical reports). The database was modelled on those of other authors [9,10]. It contains the following items: *Age of early psychomotor development*, *age at diagnosis*, *first clinical symptoms, clinical symptoms before diagnosis* (coarse facial features, recurrence of diarrhoea and otitis, umbilical or inguinal hernia, nose and throat problems including surgical procedures, hearing impairment, speech delay, hyperactivity, sleep disturbance), *evolution of neurodegenerative symptoms* (age at onset of epilepsy and treatment received, age at regression of acquired skills, age at loss of functional abilities, dysphagia, nasogastric tube/gastric button feeding), *cognitive failure through the disease* (age of normal and reduced cognitive functions and start of special education, age of partial or total loss of relationship with environment, etc.), *other significant chronological complications* (limb spasticity, kyphosis and scoliosis, and dislocated hips) and *age of death.*

The analysis of the database was conducted by a single investigator (VDCh). If the questionnaire received was not fully completed by the physician, or if the parents failed to understand some items, the investigator conducted a face-to-face or telephone interview to complete the questionnaire.

### Biochemical diagnosis

All patients included in this study were biochemically diagnosed as MPSIIIA, MPSIIIB, or MPSIIIC according to the presence of increased levels of heparan sulphate in urine and deficiency of specific enzyme activity in leukocytes or fibroblasts (sulfamidase-MPSIIIA, α-N-acetylglucvosaminidase-MPSIIIB, and heparin-α-glucosaminido-acetyltransferase-MPSIIIC). Enzymatic study of our Sanfilippo A patients showed levels < 2.5 nmol/17 h/mg in leukocytes and <50 nmol/17 h/mg in fibroblasts. For Sanfilippo B patients the levels were < 3.90 nmol/17 h/mg leukocytes and < 69 nmol/17 h/mg in fibroblasts, while for Sanfilippo C they were < 4 nmol/17 h/mg.

### Molecular genetic studies

Pathogenic mutations were previously identified in the majority of cases (Table 1). Four mutations are new and reported in this paper for the first time.

**Table 1:** Mutational analysis of MPS III patients

**Case number**	**M/F**	**Year of birth**	**MPS III subtype**	**Mutation**	**Reference**
**1**	**M**	**2004**	**III-A**	**p. [L343Pfsx158]+ p. [D235N]**	**Weber 1997 [**[21]**]**
				**(c. [1027dupC]+c. [703G>A])**	**Bunge 1997 [**[22]**]**
**8**	**F**	**1998**	**III-A**	**p. [A354P]+ p. [L343Pfsx158]**	**Monfort 1998, [**[23]**]**
				**(c. [1027 dupC] + (c. [1063G>C])**	**Weber 1997 [**[21]**]**
					**Bunge 1997 [**[22]**]**
**9**	**M**	**1996**	**III-A**	**p. [R74C +?] (c. [220C>T +?**])	**Weber 1997 [**[21]**]**
**14**	**F**	**1996**	**III-A**	**p. [R74C + R47C] (c. [220C>T + 220C>T** ])	**Weber 1997 [**[21]**]**
**15**	**M**	**2005**	**III-A**	**p. [R433Q]+ p. [R433Q] (c. [1298G>A]+**	**Chabas 2001 [**[24]**]**
				**c. [1298G>A ])**	
**17**	**M**	**1997**	**III-A**	**p. [V361fsX52)+ V361fsX52] ( c. [1079 del C + 1079delC])**	**Monfort 1998 [**[23]**]**
**19**	**M**	**2001**	**III-A**	**p. [V361fsX52 ]+p. [ R433Q] (c. [1079 del C] +c. [1298G>A])**	**Monfort 1998 [**[23]**]**
**21**	**F**	**2000**	**III-A**	**p. [S66W +?] (c. [197C>G +?])**	**Montfort 2004 [**[25]**]**
				***only Father mutation found***	
24 *	**M**	**1976**	**III-A**	**p. [V361fsX52]+ p. [V361fsX52] (c. [1079 del C] + c. [1079delC])**	**Monfort 1998 [**[23]**]**
25*	**M**	**1986**	**III -A**	**p. [V361fsX52]+ p. [V361fsX52] ( c. [1079 del C] + c. [1079delC])**	**Monfort 1998 [**[23]**]**
**26**	**F**	**1990**	**III-A**	**p. [S66W ]+ p. [R74H]**	**Montfort 2004 [**[25]**]**
				**(c. [197C>G] + c. [220C>T])**	**Weber1997 [**[21]**]**
27 **	**M**	**1981**	**III-A**	**p. (V361fsX52)+ p. (R206P) (c. [1079 del C]+ c. [617G>C])**	**Monfort 1998 [**[23]**]**
					**Montfort 2004 [**[25]**]**
28**	**M**	**1984**	**III-A**	**p. (V361fsX52)+ p. (R206P) (c. [1079 del C]+ c. [617G>C])**	**Monfort 1998 [**[23]**]**
					**Montfort 2004 [**[25]**]**
**36**	**F**	**2007**	**III-A**	**p. [R74C]+ p. [Q85R]**	**Weber1997 [**[21]**]**
				**(c. [220C>T] +c. [254A>G])**	**Montfort 1998 [**[23]**]**
**37**	**F**	**2009**	**III-A**	**p. [V361SfsX52] + p. [V361fsX52] (c. [1079 del C] +**	**Montfort 1998 [**[23]**]**
				**c. [1079 del] )**	
**39**	**M**	**2005**	**III-A**	**p. [V361fsX52)+ p. [V361fsX52] c. [1079 del C] + c. [1079delC])**	**Monfort 1998 [**[23]**]**
**42**	**M**	**2002**	**III-A**	**p. [F1625fsX6] +p. [ R206P]**	***Not previously reported***
				**c. ([484–486 del TC] + c. ([617G>C ])**	**Montfort 1998 [**[23]**]**
**43**	**M**	**1991**	**III-A**	**p. [R433 W]+p. [R433W] (c. [12097C>T] + c. [1297C>T])**	**Beesley 2000 [**[26]**]**
44***	**F**	**1973**	**III-A**	**p. [V75RfsX116; V501M] (c. [221insC; 1501G>A]**	
45***	**F**	**1976**	**III-A**	**p. [V75RfsX116; V501M] (c. [221insC; 1501G>A]**	
**46**	**F**	**2008**	**III-A**	**p. [L343Pfsx158 ]+ p. [L343Pfsx158]**	**Monfort 1998 [**[23]**]**
				**(c. [1027insC]+c. [127insC])**	**Weber 1997 [**[21]**]**
**52**	**F**	**1971**	**III-A**	**p. [R74H +?] (c. [221G>A + ?])**	**Bunge 1997 [**[22]**]**
**55**	**M**	**2010**	**III-A**	**p. [V361fsX52]+p. [S66W] (c. [.1079c >G] c. [197C>G])**	**Montfort 1998 [**[23]**] Montfort 2004 [**[25]**]**
**2**	**F**	**2004**	**III-B**	**p. [R38W]+ p. [R38W ] (c. [112C>T] + c. [112C>T])**	**Beesley 2005 [**[27]**]**
**3**	**F**	**2001**	**III-B**	**p. [R234C + L622P] +**	**Beesley 1998 [**[28]**]**
				**c. (700 C >T+1865 >C )**	***Not previously reported***
**10**	**F**	**2004**	**III-B**	**p. [M338V] + [W404X+R541W]**	***Not previously reported***
				**(c. [1012A>G]+[1211G>A+1621C>T])**	**Bunge 1999 [**[29]**]**
**18**	**M**	**2002**	**III-B**	**(c. [531+5G>A] +c. [531+5G>A]) IVS2+5G>A + IVS2+5G>A**	***Not previously reported***
**31**	**M**	**1991**	**III-B**	**(c. [531+5G>A ]+ c. [531+5G>A]) IVS2+5G>A + IVS2+5G>A**	***Not previously reported***
**32**	**F**	**2000**	**III-B**	**p. [R234C]+p. [W168X]**	**Beesley 1998 [**[28]**]**
				**(c. [700C>T+503G>A])**	**Coll 2001 [**[30]**]**
**35**	**F**	**1990**	**III-B**	**p. [R643C+P115S](c. [1927C>T+ 343C>T])**	**Beesley 1998 [**[28]**]**
					**Schmidtchen 1998 [**[31]**]**
**23**	**M**	**2003**	**III-C**	**C. [372-2A>G + 372-2A>G]**	**Canals 2011 [**[17]**]**
				**(IVS3-2A>G + IVS3-2A>G)**	
**33**	**M**	**1987**	**III-C**	**c. [234+1G>A] + c. [234+1G>A] (IVS2 + 1G>A + IVS2+1G>A)**	

M: Male F: Female.

*24 -25 / ** 27 – 28 / *** 43 – 44: were two pairs of siblings from different families.

* 24 – 25 death at 19 and 26 years respectively.

### Statistical analysis

SPSS version 12.0 for Windows (SPSS Inc, Chicago, IL) was used for data processing. Survival curve estimates were calculated using the Kaplan-Meier product-limit method, and the resulting curves were compared between groups using the log-rank test. The age of onset of each psychomotor impairment was taken as survival data (in the absence of this information, data corresponded to the age of the last visit or death).

### Ethical aspects

Written informed consent was obtained from all parents or legal guardians of the children involved in this study. Additional consent was obtained from parents to review the child’s medical history. To preserve patients’ privacy, each child was assigned a study number, which was then used to process data. This study was formally approved by the local ethics committee.

## Results

Data were collected from 55 Spanish patients: 34 MPS III A (62%), 11 MPS IIIB (20%), and 10 MPS IIIC (18%). There were 28 females (51%) and 27 males (48%). The median age of patients at the time of data collection was 13 years (range 2.7- 41 years).

### Early psychomotor development

The ability to sit down unassisted was acquired between 6 and 8 months (normal range), and walking independently between 12 and 20 months of age in all patients (normal range). First single words were reported in 51 patients and were acquired after 18 months median age. (normal range 12–18 months old). Partial sphincter control (feces and urine) was reported in 52 patients and was acquired only in 44% of patients with a median age of 3.2 years old (normal range between to 2–3 years).

### First clinical symptoms before diagnosis

**Figure 1:**
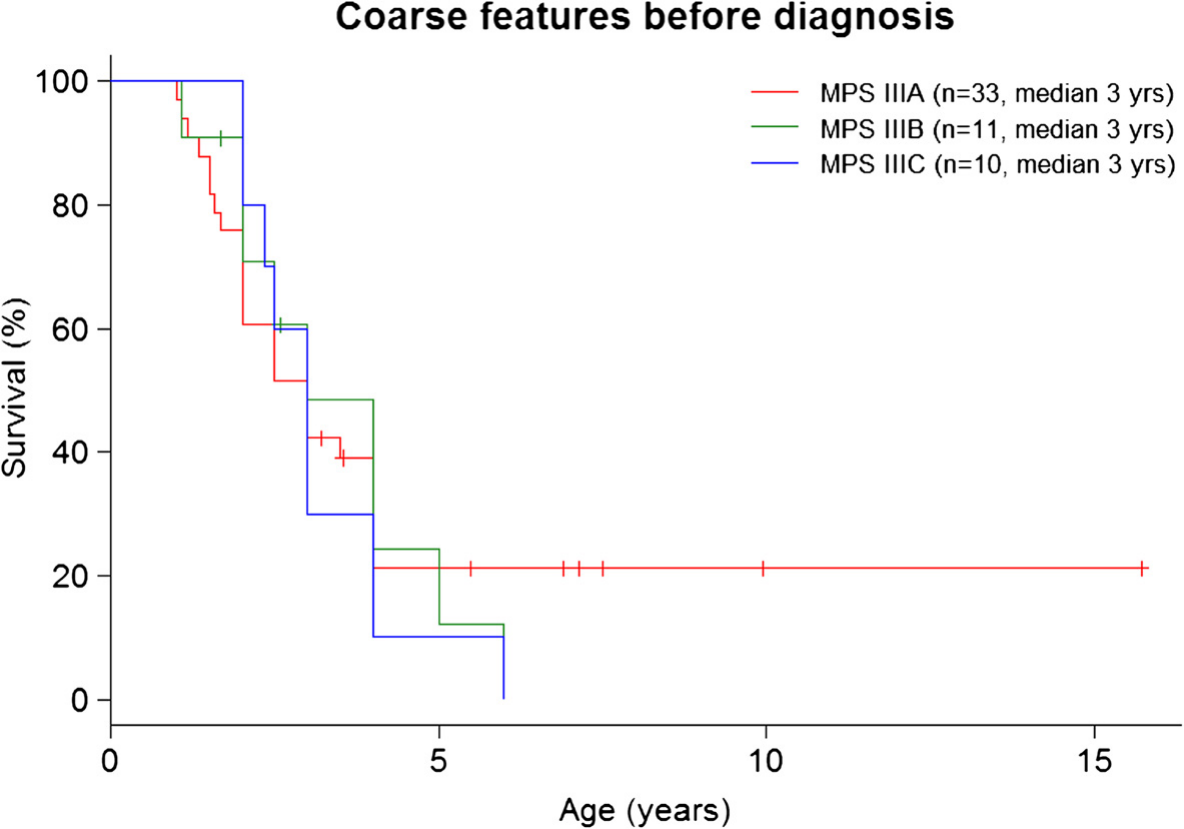
Coarse features before diagnosis.

**Figure 2:**
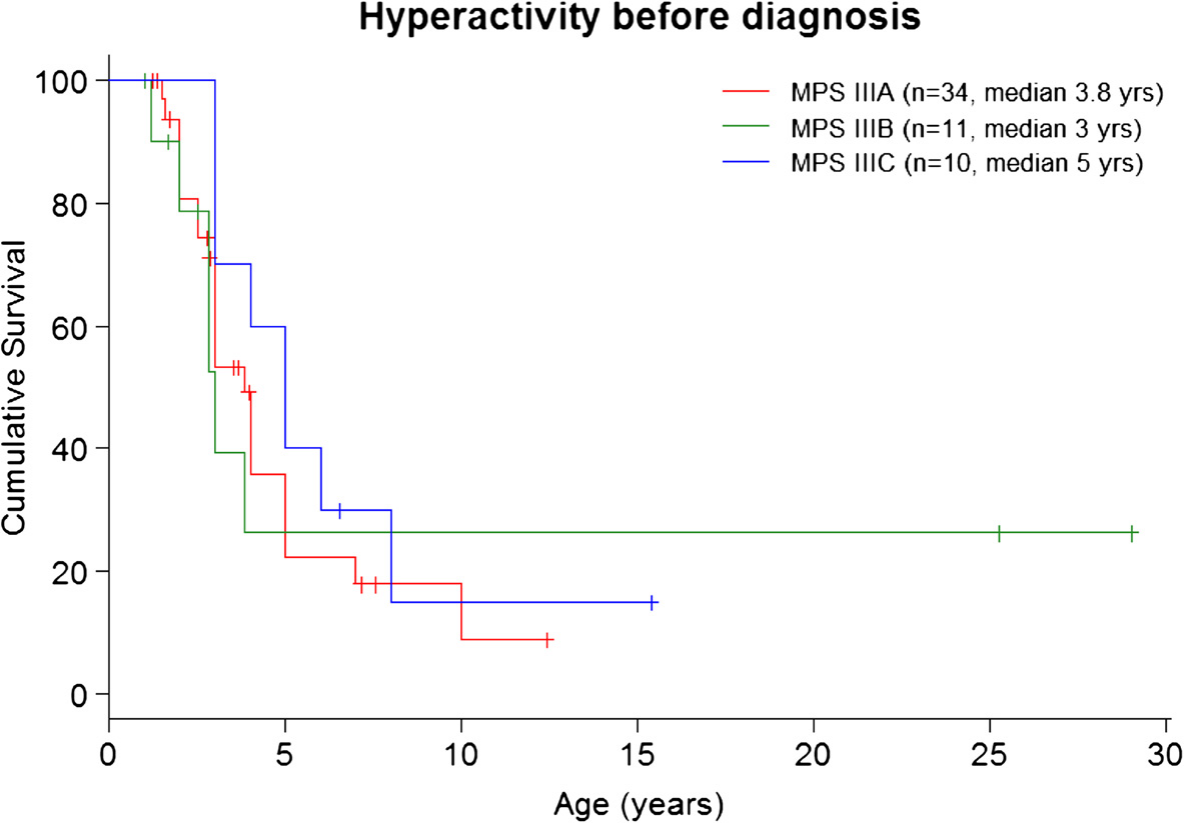
Hyperactivity before diagnosis.

Diagnosis in the different subtypes are shown in Table 2. The most frequent symptoms were delayed speech in 85% of MPS III, followed by coarse facial features in 78%, and hyperactivity in 65% of cases at mean age of 3 years old Figures 1 and 2. Other frequent non-specific symptoms were recurrent diarrhoea in 50% and recurrent otitis in 46% of patients during the first two years of life.

**Table 2:** Symptoms before diagnosis in MPS III Subtypes

**Symptoms before diagnosis**	**MPS Subtype (N°)**			**N° casos**		**Median**
	**A**	**B**	**C**			**Age**
						**(Range)**
**Coarse facial features**	**24**	**9**	**9**	42	**78%**	3 years
						( 1 – 4 y)
**Recurrent diarrhea**	**17**	**4**	**6**	27	**50%**	2 years
						(1 – 4.5 y)
**Umbilical hernia**	**7**	**4**	**1**	12	**22%**	1 months
						(0 – 8 months)
**Inguinal hernia**	**4**	**0**	**0**	4	**7%**	1,6 year
						( 0–3 y)
**Recurrent otitis**	**15**	**5**	**5**	25	**46%**	1 year
						(0–3.5 y)
**Hearing loss**	**12**	**2**	**6**	11	**30%**	2.2 year
						(1.5 – 6 y)
**Adenoidectomy**	**14**	**2**	**7**	23	**43%**	3 year
						(2 – 6 y)
**Tonsillectomy**	**8**	**3**	**4**	15	**28%**	3.8 year
						(2 – 9 y)
**Speech delayed**	**27**	**11**	**8**	46	**85%**	3 year
						(2.5 – 7 y)
**Hyperactivity**	**21**	**6**	**8**	35	**65%**	3 year
						(1.5 – 10 y)
**Sleep disorders**	**14**	**5**	**5**	24	**44%**	1 year
						( 2 months -6 y)

### Median age at diagnosis

For MPS IIIA subtype was IIIA 4.4 years (1.2 – 16 years), for IIIB 3.1 years (1–29 years), and for IIIC 6.3 years (3.4-22 years).

### Evolution of neurodegenerative symptoms

**Figure 3:**
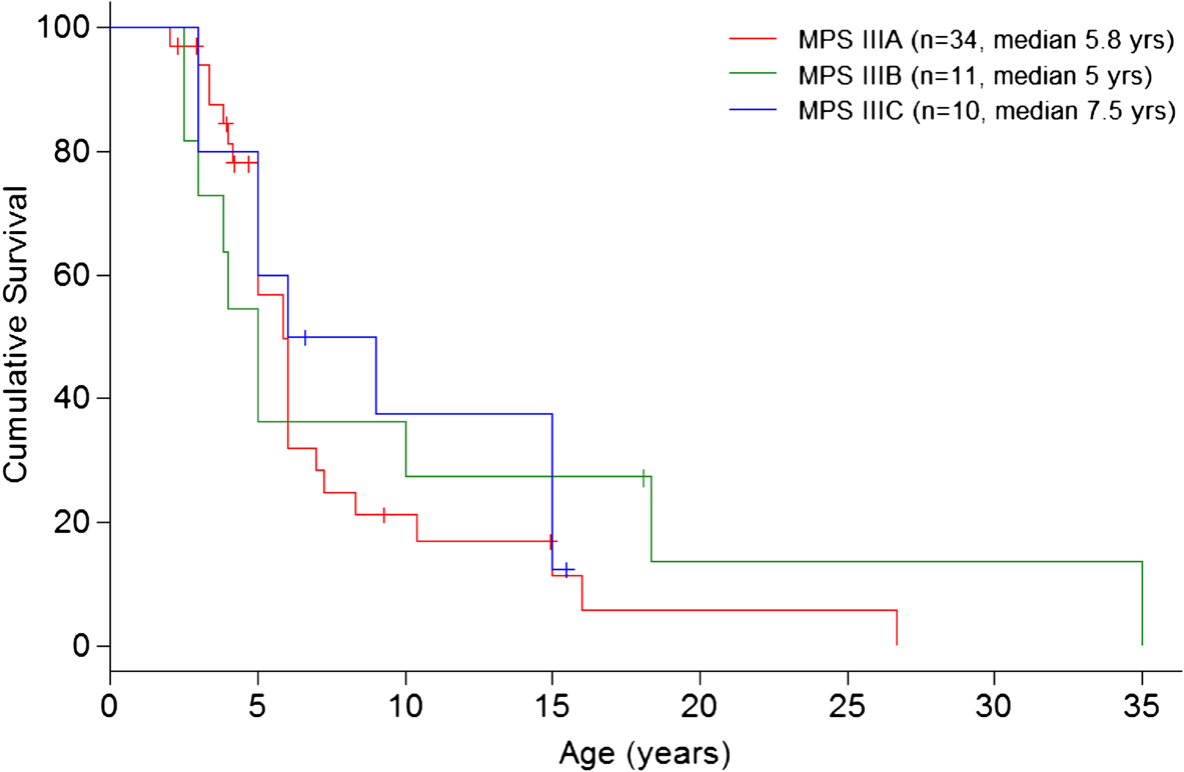
Age of speech loss.

**Figure 4:**
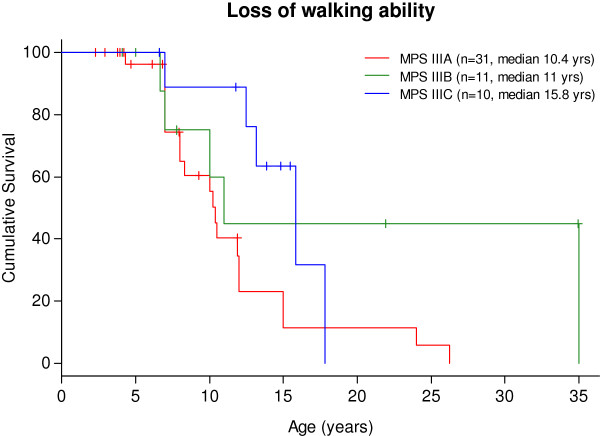
Loss of walking ability.

45% of patients presented *epilepsy* with a median age of 8.7 (2.5 – 37) years old: in MPS IIIA at 7 (2.5–16), in MPS IIIB at 12.5 (5.5–37), and in MPS IIIC at 10.4 (5–16.5) median years old. Convulsions were mainly generalised tonic-clonic seizures and were usually well-controlled with one or two antiepileptic drugs (data not shown). *Age of loss of speech* in different MPS III subtypes is shown in Figure 3. *Clumsiness in walking* started at a median age of 7.5 years old (2.5 – 25): in MPS IIIA at 7 (2.5 – 25), in MPS IIIB at 7.5 (3 – 22.5), and in MPS IIIC at 9 (4.5 - 15) years of age. Data about *losing walking ability* are reported in Figure 4. The median age of *loss of unassisted sitting* was 11.5 years old (5 – 33): in MPS IIIA it was 10.5 years, in MPS IIIB 14 years, and in MPS IIIC 13.5 years old. *Dysphagia* started at a median age of 10.8 years old in MPS IIIA and 13.6 years old in MPS IIIC. Data on MPS IIIB were not available. *Button gastric feeding* was required in 10/34 patients, mostly subtype A with an average age of 13 years old. *Cardiac examinations showed valvular disease in only one patient MPSIIIA at 3 years old and slight cardiomyopathy has been detected only in four patients (2 patients at 3 years, one at 3 years and 6 months and one at 10 years of age).*

### With respect to cognitive failure through the disease

**Figure 5:**
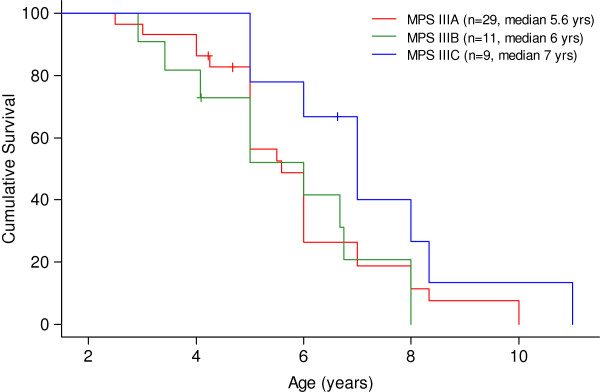
Special school for mentally retarded.

The start of special schooling was encountered at a median age of 6.2 years of age (Figure 5). Only 14 parents remembered their children managing to write their own name, and that was between 4 and 6 years of age. Different neurocognitive studies were done on only a few patients, so we could not compare outcomes. The median age of onset of losing relationship with environment in MPS subtypes was 7 years old in MPS IIIA, 8.3 years old in MPSIIIB, and 9.7 years old in MPS IIIC.

### Other significant chronological findings

(Median age) were limb spasticity in 67% (8.3 years old), kyphosis and scoliosis in 52% (8 years old), and dislocated hip in 11% (9 years old).

Nine MPS IIIA (two pairs of siblings) and 1 MPS IIIB patients had died before the study started. The median *age of death* for MPS IIIA patients was 15 years (11.5 – 26 years); the figure was 19 years for the one MPS IIIB. The cause of death was respiratory infection in 6 patients and cardiorespiratory failure in 4 patients.

### Mutational analysis

Genotypes of patients are shown in Table 1. As can be seen, the majority of mutations were previously described except for 4 different mutations which appear in this paper for the first time. Three of them were missense mutations found in three different MPSIIIA patients in compound heterozygosis (p.L622P, p.M338V and p.R541W). The fourth was a splicing mutation (IVS2+5G>A) found in an MPSIIIB homozygous patient.

## Discussion

This retrospective study, based on 55 patients with Sanfilippo disease, is the first extensive Spanish clinical description of the disease. The registry included patients born between 1971 and 2010 and is therefore heterogeneous in terms of age group. No significant differences were found in gender.

MPS IIIA is the most common type of Sanfilippo in the Spanish population (62%), probably due to population migration from central Europe.

These findings contrast with previous retrospective studies which found MPS IIIA to be the most frequent type of Sanfilippo disease in northern Europe [19,32-34], whereas MPS IIIB is more frequent in southern Europe [35-38].

Due to differences in the number of patients in each subtype, it was difficult to establish comparative statistical studies (MPS IIIA: 62%, MPS IIIB: 20% and MPS IIIC: 18%).

In the early psychomotor development of patients with MPS IIIA, B, and C, the acquisition of sitting and unassisted walking were within normal parameters, as previously described [4,5]. At 18 months of mean age, mild speech delay, without any new words was evident in most patients, corresponding to the onset of the first phase of the disease [2,4,5,19].

The median age of diagnosis was under 5 years in MPS III A and MPS III B, and over 5 years in MPS C. These results were similar to those previously described for MPS IIIA [10,20], MPS IIIB [20], and MPS IIIC [11]. Diagnostic delay was common, particularly in patients with a slow progression or attenuated phenotypes, especially in MPS IIIC [5,11].

The age of diagnosis is quite delayed in our group of patients if we consider disease modifying therapy, such as gene therapy and enzyme replacement therapy as promising future curative treatments, which highlights the need for early diagnosis for family genetic counselling. We should emphasize that in our series we found 5 pairs of siblings.

**Figure 6:**
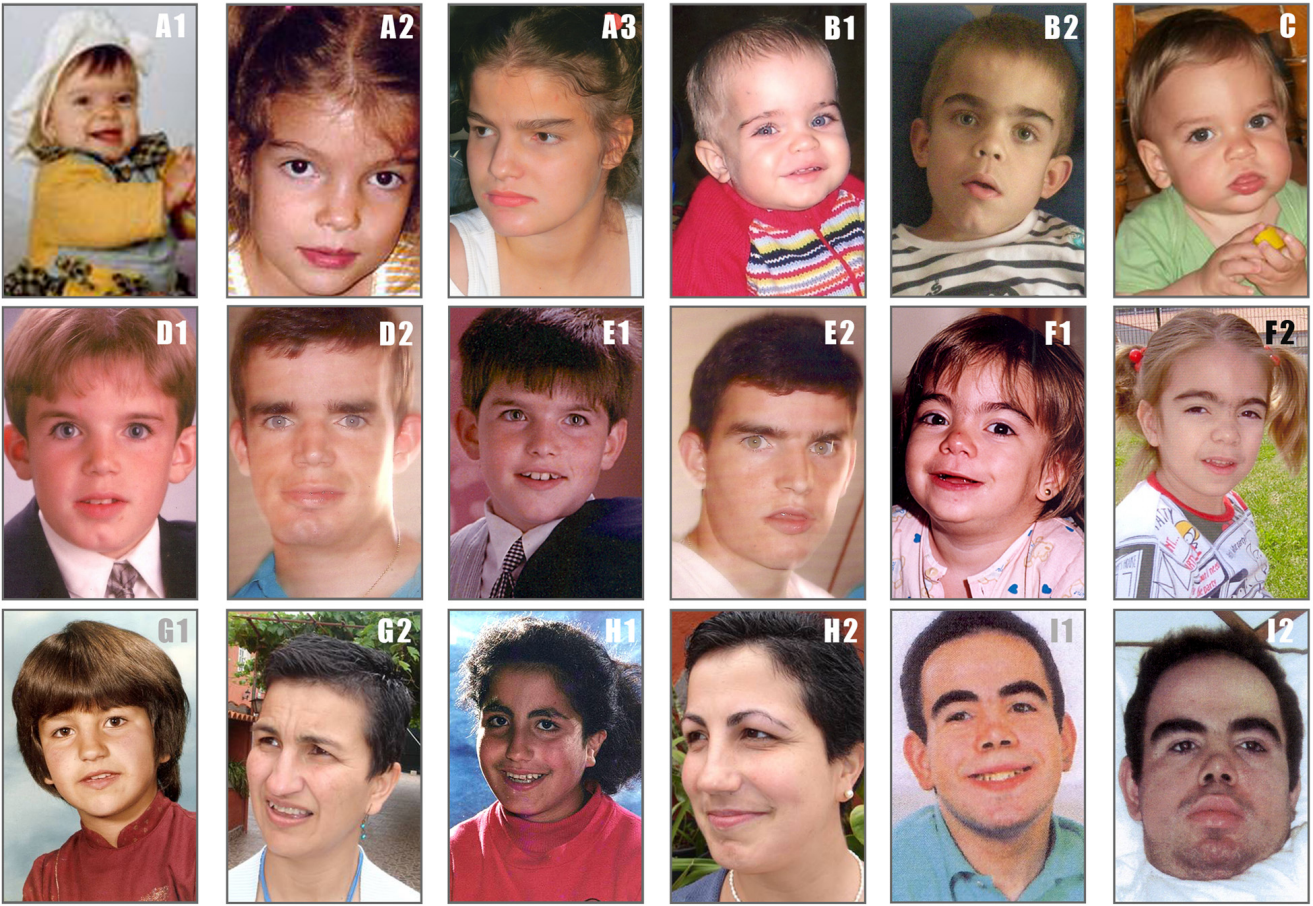
**Some facial pictures from Sanfilippo patients who participated in this study.** We observed variability facial features at different age in the same patients except in picture C (only one picture). The MPSIII subtype and age at patients are as follows: **(A)** Female MPS IIIA A1 -10 months, A2 -6 years and A3 -14 years. **(B)** Male MPS IIIA B1 -12 months, -7 years. **(C)** Male MPS IIIA 15 months. **(D)** Male MPS IIIA D1-10 years, D2-25 years. **(E)** Male MPS IIIA E1 -10 years, E2- 22 years. **(F)** Female MPS IIIB F1-18 months, F2- 6 years. **(G)** Female MPS IIIB G1 7 years, G2 39 years. **(H)** Female MPS IIIB H1-14 years, H2 -36 years. **(I)** Male MPS IIIC I1-12 years, I2 -24 years.

The most frequent clinical symptom before diagnosis in our series was speech delay, as previously described [9,10,20]. Coarse facial features were present before diagnosis in 78% of MPS III patients in our series, in contrast with previous reports in which facial dimorphisms appeared later [9,10]. Although facial dimorphisms can be easily discernible in MPS I–Hurler or MPS II-Hunter syndrome [39], patients with MPS III often have early very mild coarse facial features [4,5], as we found from pictures submitted by families at different ages (Figure 6). However in the photos corresponding to two pairs of siblings MPS IIIA (Figure 6 D1-D2 and E1-E2) and MPS IIIB (Figure 6 G1-G2 and H1-H2) with mild phenotype, we see that coarse facial features appeared much later, in the third and fourth decades of life, respectively. And even in our series we can see that in two MPSIIIB sisters at 36 and 39 years of age coarse features had not appeared. (Figure 6 G2 – H2).

Behavioural problems such as hyperactivity are very frequent among Sanfilippo patients from the age of three, heralding the onset of the second phase of the disease [4-6,8]. Behavioural problems worsened with sleep disturbances which seemed to start during the first year of life in our series. These problems remained and were enhanced during the first decade of life, and their management is very difficult [9-11,40,41].

Other frequent, miscellaneous, non-specific symptoms during the first two years of life were recurrent diarrhoea, recurrent otitis, and hearing impairment, as described by other authors [4,11].

Regarding neurodegenerative clinical symptoms, epilepsy was present in almost half of the MPSIII. Most MPS IIIA patients manifested epilepsy before age 10, whereas for MPS IIIB and MPS IIIC this occurred at a later age. Only one MPS IIIB patient started epilepsy in the third decade of life. These results were comparable to those described previously [9,20]. We found predominantly generalized seizures occurring in the second or third phase of the disease and these were usually well-controlled with one or two drugs.

As with many degenerative diseases, loss of speech preceded loss of motor functions, marking the beginning of the regression of psychomotor skills [2,9,10]. Loss of speech takes place at around 5 years of age in MPS IIIA and MPS IIIB, and later on in MPS IIIC [11,42]. Patients start losing sentences and afterwards utter single words with slurred speech, eventually arriving at a state marked by the absence of verbal language.

Based on parents’ reports, the majority of patients never developed complete expressive language.

In our series, clumsiness in walking preceded by 4 years the loss of independent walking in MPS IIIA and MPS IIIB, whereas it was delayed by almost 7 years in MPS IIIC. Walking was lost in MPS IIIC at a median age of 15.8 years in our study and between the ages of 20 and 30 in a sample from the Netherlands [11]. These findings indicate that the deterioration of motor function in MPS IIIC starts significantly later compared to other Sanfilippo subtypes [11,42]. Loss of independent walking and consequent outdoor wheelchair use is a dramatic change in the life of patients and their families.

The onset of dysphagia parallels the loss of motor milestones in MPS IIIA and MPS IIIC. The need for a gastric feeding button marks the late stage of the disease; in our study 10/34 patients, mostly subtype A, required this in their second decade of life.

Enrolment in special education programs for people with learning disabilities from the beginning of primary school indicates an evident progressive cognitive failure. In this retrospective study, the cognitive decline was difficult to assess due to the diversity of psychometric tests performed on each patient. In an interesting cognitive development assessment of an MPS III group from the Netherlands (A, B, and C) patients showed broad variation in intellectual disability [6].

Onset of loss of relationship with the environment was established 2 or 3 years after speech loss among Sanfilippo patients. In the third stage of the disease, the full loss of relationship with the environment was preceded by the cessation of behavioural disturbances, as was previously reported at 12.5 years average age in a group of MPS IIIA [9,10].

Orthopaedic manifestations, such as the kyphosis and scoliosis found in 50% of our patients, tend to appear in the second phase of the disease [43].

On the basis of previous reports, death in Sanfilippo patients occurs at the end of the second decade. Longer survival was reported in MPS IIIA attenuated phenotype (Valstar [9]). In our group of MPS IIIA patients, the median age of death was 15, and respiratory infections were the main cause of death.

Sanfilippo syndrome is an example of lysosomal diseases in which there is not a complete genotype-phenotype correlation but in some countries a substantial relation has been observed [9,20].

Based on the new missense mutations presented in this paper we think that they are the cause of the disease because they affect a conservative amino acid and/or are predicted as pathogenic by bioinformatic programs as polyphen.

In the case of the new splicing mutation found in case 18, we need to study the cDNA to confirm its pathological effect.

As a final point, not all the patients in the study underwent molecular genetic study, since this is a retrospective analysis that includes a heterogeneous group of patients. Those born between 1970 and 1980 were less likely to have DNA in the bank and thus to undergo mutational study.

The diversity in 1clinical manifestations is due to allelic heterogeneity [37]. A large number of mutations in each subtype, MPS IIIA, IIIB, and IIIC, have been reported. [16,22,23,27,28,30,31,44],[45].

An international registry with larger patient populations and molecular studies may help to establish the spectrum of clinical phenotypes and genotypes in each subtype of MPSIII.

## Conclusions

MPS IIIA is the most frequent subtype in Spanish Sanfilippo patients.

We support the idea that children with non-specific developmental delay, and especially speech delay, behavioural abnormalities and/or mild coarse facial features, should be tested for MPS III, as well as patients without coarse features that may develop attenuated phenotypes.

We stress the importance of learning about the natural history of Sanfilippo disease to determine not only the prevalence and/or incidence of the disorder in each country, but also to learn the best time to establish short-term treatment with gene therapy, which may change the prognosis of this disease.

## Competing interests

We have no competing interests to declare. This study was supported by a grant to VD from the Spanish Society of Mucopolysaccharidosis Disease and Related Syndromes (MPS España).

## Authors’ contributions

All authors participated in drafting the manuscript. MP: coordinator of the study. MP and VD: designed the study. VD: collected the database and statistical analysis. MO: participated in statistical analysis. MJC: carried out the molecular genetic studies. LG: participated in the biochemically diagnosed. All authors read and approved the final manuscript.
